# Bioconjugates of Toluidine Blue Derivatives with Human Serum Albumin and Their Complexes with Cucurbit[7]uril as Drug Delivery Vehicles for Photodynamic Therapy

**DOI:** 10.1021/acsomega.5c03731

**Published:** 2025-10-27

**Authors:** Nory Mariño-Ocampo, José Robinson-Duggon, Daniel Zúñiga-Núñez, Daniel Guerra Díaz, Belinda Heyne, Denis Fuentealba

**Affiliations:** † Laboratorio de Química Supramolecular y Fotobiología, Departamento de Química Física, Escuela de Química, Facultad de Química y de Farmacia, 28033 Pontificia Universidad Católica de Chile, Vicuña Mackenna 4860, 7820436 Santiago, Chile; ‡ Facultad de Ciencias Naturales, Exactas y Tecnología, Departamento de Bioquímica, 54717 Universidad de Panamá, Panamá 0824-00110, República de Panamá; § Sistema Nacional de Investigación (SNI), Secretaría Nacional de Ciencia, Tecnología e Innovación (SENACYT), Panamá 72700, República de Panamá; ∥ Departamento de Química Orgánica, Facultad de Ciencias, 14655 Universidad de Chile, Las Palmeras 3425, 7800003 Santiago, Chile; ⊥ Department of Chemistry, 2129 University of Calgary, 2500 University Drive NW, T2N 1N4 Calgary, Alberta , Canada

## Abstract

Photodynamic therapy (PDT) is a promising cancer treatment. The present work used two toluidine blue O (TBO) derivatives covalently conjugated to human serum albumin (HSA) as a drug delivery system and bound to cucurbit[7]­uril (CB[7]) as a protective nanocapsule in order to study their effect of cellular uptake and phototoxicity in HeLa cells. This study explored covalent and noncovalent interactions with the delivery systems, including HSA-PS@CB[7] complexes, to optimize PDT efficacy. Supramolecular complexes between TBO derivatives and CB­[7] exhibited high binding affinities, significantly improving the photophysical properties of the photosensitizers. In vitro studies in cancer cells demonstrated that HSA covalently bound to TBO derivatives led to significant increases in cellular uptake and was strongly influenced by the type of conjugation (disulfide bond versus thia-Michael adduct). The results suggest that combining the properties of HSA as a carrier and CB[7] as a protective nanocapsule could be exploited for improved PDT applications.

## Graphical abstract

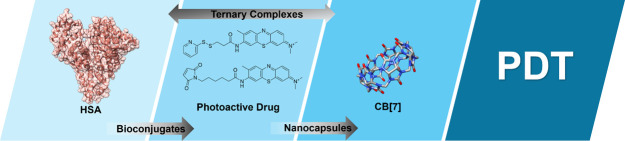

## Introduction

Cancer represents the second cause of death worldwide.^1^ As the search for treatment alternatives continues, photodynamic therapy (PDT) arises as a minimally invasive procedure, which involves a photoactive drug (PS), light, and molecular oxygen. Most PS corresponds to naturally occurring or synthetic chromophores that are incorporated into cancerous cells and absorb light in the red or infrared regions of the spectrum. The mechanism of action of PDT starts with the administration of PS, followed by their accumulation in cancer cells and activation by light, where electronic transitions occur. Upon light activation, a PS in its ground state transitions to a short-lived singlet excited state (^1^PS) and then to a longer-lived triplet excited state (^3^PS). It is this triplet excited state that leads to the generation of reactive oxygen species (ROS) via type I and/or type II reactions.^2^ In type I reactions, electron transfer or hydrogen abstraction from biological substrates leads to the formation of radical and nonradical reactive species such as superoxide (O_2_^·–^), hydroxyl (OH^·^), and hydrogen peroxide (H_2_O_2_). In type II reactions, energy transfer from the PS to molecular oxygen generates singlet oxygen (^1^O_2_). All of these ROS can trigger cancer cell death by necrosis and/or apoptosis depending on the subcellular localization of the PS.^2,3^

Recent research efforts aim to optimize PS to address challenges such as low solubility in aqueous solutions and lack of selectivity toward cancer cells.^4^ Among second-generation PS, toluidine blue O (TBO) appears as a promising candidate for PDT.^5,6^ TBO is an inexpensive cationic phenothiazine derivative that has demonstrated low toxicity and excitation at a relatively low energy (626 nm). Additionally, its singlet oxygen quantum yield (Φ_Δ_) can be relatively high.^7^ TBO also demonstrates a high affinity for nucleic acids, lipopolysaccharides, and cell membranes.^8,9^

On the other hand, drug delivery systems (DDSs) have been extensively explored in PDT to overcome challenges such as low solubility, poor selectivity, and low incorporation into cancer cells. Common DDSs used in PDT span from nanoparticles and liposomes, to peptides, proteins, and macrocycles.^4^ However, drug–protein interactions occur naturally after drug administration and are particularly important for drug biodistribution and elimination from the body.^10^ After intravenous administration, many drugs have a high degree of binding with serum proteins, with albumin being the most important one.^11^

Indeed, human serum albumin (HSA), the most abundant protein in blood plasma (∼60%), plays a key role in drug delivery due to its multiple binding capabilities. With a molecular mass of 66.4 kDa, HSA contains three homologous domains, each with two subdomains, and has two binding sites (Sudlow’s I and II) and one free cysteine residue (Cys34). This Cys34 residue has been exploited for covalent drug conjugation and drug delivery purposes.^12^ HSA conjugation aids in cancer cell uptake by the enhanced permeation and retention effect (EPR) as well as specific receptors like gp60 and SPARC.^13^

Other DDSs found in the literature are macrocycles from the curcubit­[*n*]­uril family (CB­[*n*], *n* = 5–8,10) because they exhibit low cytotoxicity, high binding affinities with drugs, and good solubility in physiological media, enabling drug stabilization while improving drug delivery.^14,15^ This family of macrocycles is synthesized via the condensation of glycoluril units in the presence of paraformaldehyde under acidic conditions.^16^ Furthermore, CB­[*n*]-based inclusion complexes can significantly enhance the photophysical and photochemical properties of PS such as fluorescence, photostability, and singlet oxygen generation, increasing the use of these macrocycles in PDT.^17,18^

In a previous work, we explored the covalent conjugation of TBO derivatives to HSA through disulfide bonds and thia-Michael addition.^19^ Thus, two sets of TBO derivatives were prepared bearing a disulfide bond, which allowed disulfide exchange reaction with Cys34, or a maleimide, which reacts rapidly with Cys34 through thia-Michael addition. On the one hand, the disulfide bond is reversible, allowing for this derivative to be released intracellularly. On the other hand, the thia-Michael adduct formed upon reaction of the maleimide with Cys34 is expected to be more stable under physiological conditions. Despite multiple advantages as a DDS, HSA quenched the fluorescence emission and singlet oxygen generation of the PS.^19^ To address this drawback, we also used macrocycles from curcubit­[*n*]­uril as a protective nanocapsule.

As mentioned above, CB[7] can influence the photophysical and photochemical properties of PS, including enhanced fluorescence and singlet oxygen quantum yield, which can be advantageous for PDT applications.^17^

Previously, we have explored the combination of these DDSs to enhance PDT applications using TBO fatty acid derivatives, which are bound noncovalently to HSA.^5,20^ In the present work, we evaluated the PDT efficiency of two TBO derivatives with disulfide and thia-Michael reacting groups,^19^ covalently bound to HSA and in combination with CB[7] as a protective nanocapsule (Scheme 1).

**Scheme 1:**
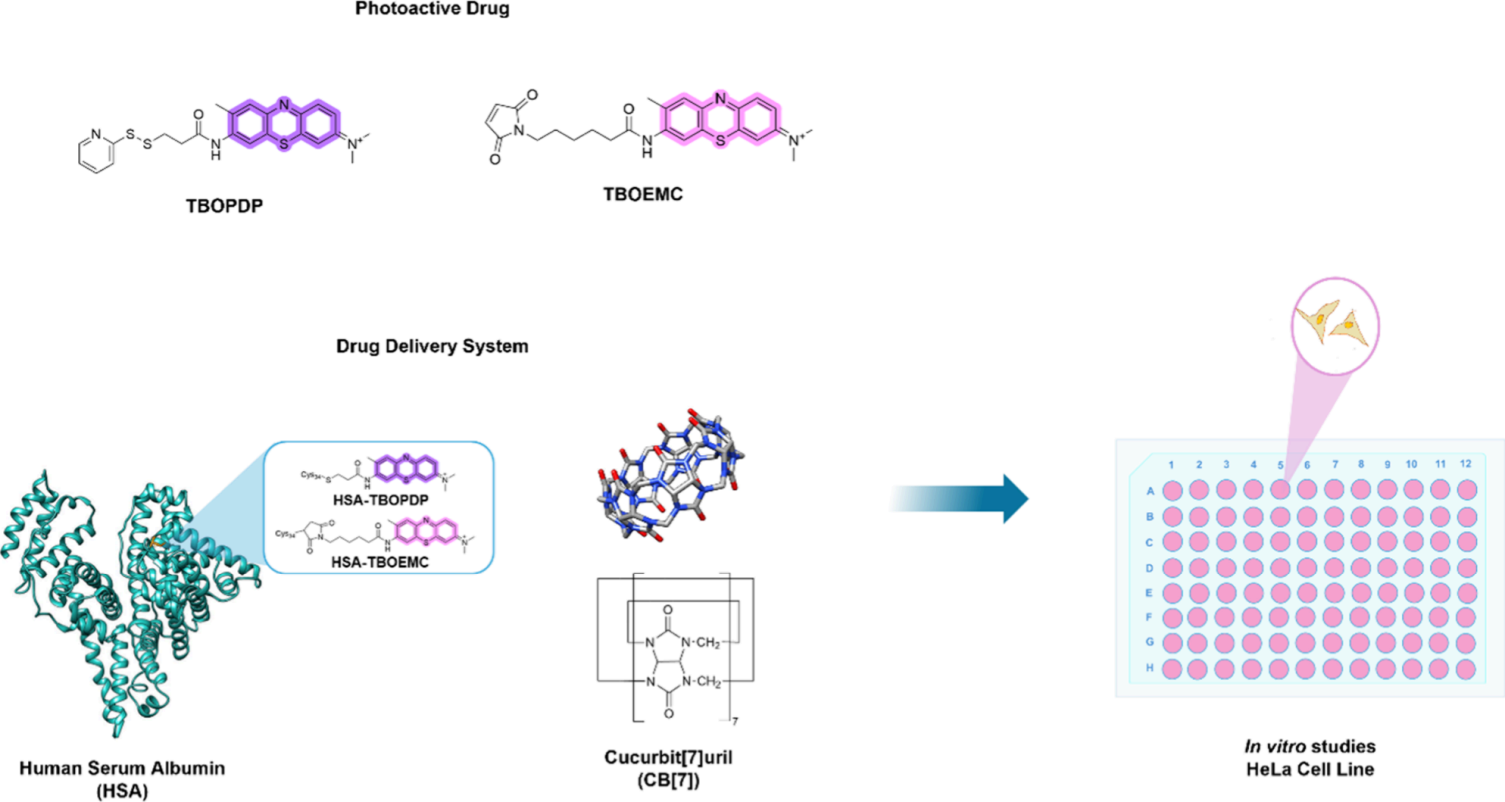
Structures for the PSs (TBOPDP and TBOEMC) and Drug Delivery Systems (HSA and CB[7]) Used in This Work

## Results and Discussion

### Absorption and Fluorescence Measurements

The synthesis and photophysical properties of TBO derivatives, TBOPDP and TBOEMC, as well as their covalent conjugation with HSA, have been previously reported.^19^ In this study, first we evaluated the binding effects of CB[7] to the TBO derivatives (Scheme 1). Toluidine blue exhibits a low fluorescence quantum yield in phosphate buffer (DOI: 10.1111/php.14066), a property also observed for its derivatives TBOPDP and TBOEMC, which is reflected in their weak emission intensity. Upon complexation with CB[7], both TBO derivatives exhibited hypsochromic shifts and a slight enhancement of their fluorescence spectra (Figure 1). These shifts are attributed to the CB[7] cavity, which provides a low polarizability environment, thereby influencing the physical and chemical properties of the guest molecules.^15,21^

**Figure 1:**
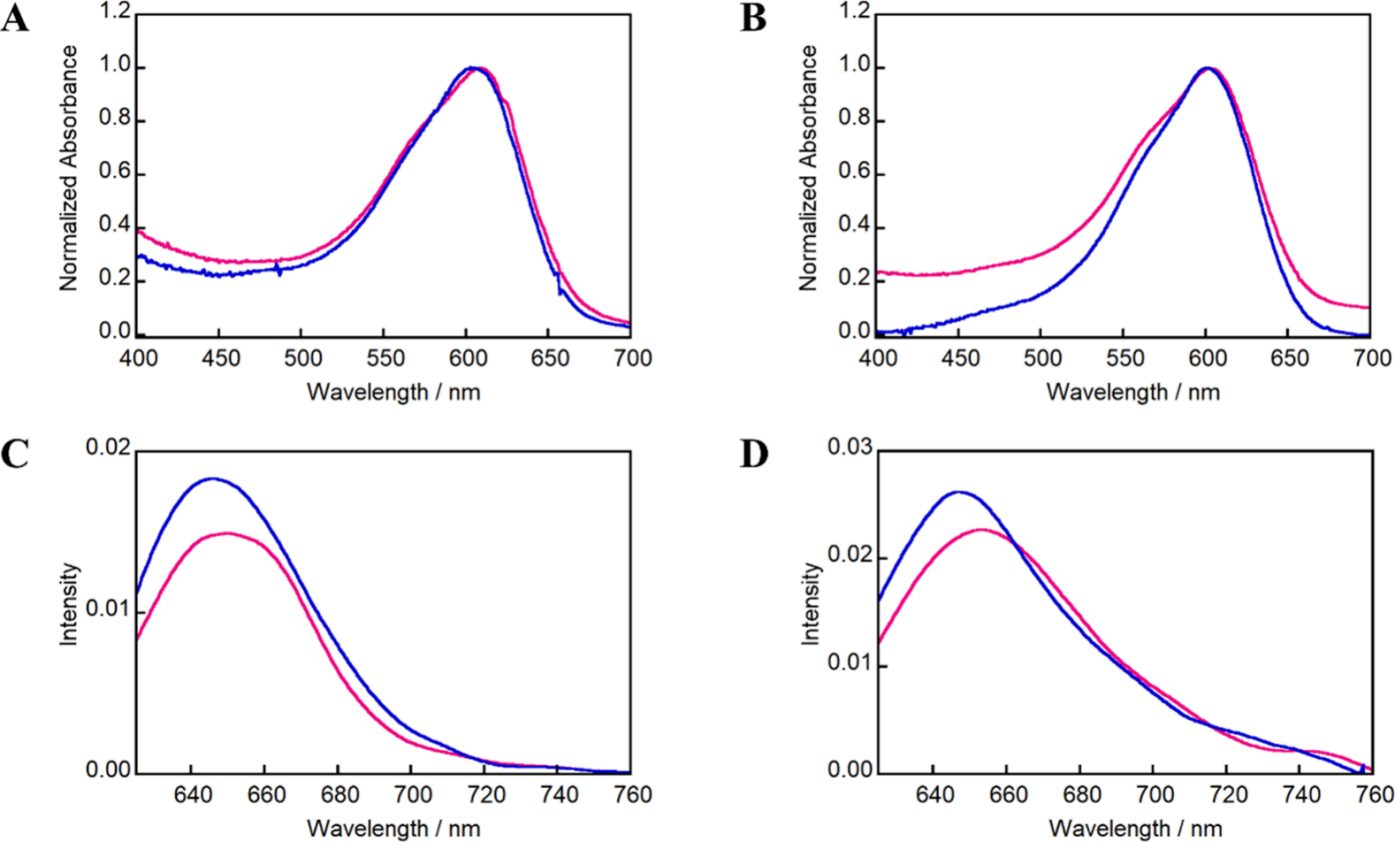
Absorption spectra of (A) TBOPDP (2 μM) and (B) TBOEMC (2 μM) recorded in the absence (magenta) and presence (blue) of 50 μM CB[7]. Emission spectra of (C) TBOPDP (2 μM) and (D) TBOEMC (2 μM) under the same conditions. Emission spectra were obtained with excitation at the absorption maxima by using a 10 mM phosphate buffer (pH 7.0) as the solvent. The fluorescence intensity corresponds to arbitrary units.

The average fluorescence lifetime of TBO, as well as its derivatives TBOPDP and TBOEMC, increased when complexed with CB[7] and HSA (Table 1), compared to the free PS. This increase in lifetime is attributed to the mobility restriction of the PS within the CB[7] cavity and the microenvironment around Cys34 in HSA, which reduces nonradiative deactivation pathways, such as energy transfer with the solvent.

**Table 1:** Photophysical Properties of TBO Derivatives and Covalent HSA-TBO Derivatives Free or Complexed with CB[7] in Phosphate Buffer

complex	λ_abs_/nm	λ_em_/nm	⟨**τ**_f_⟩**/ ns**	τ_Δ_/ μs	**Φ**_Δ_ ^a^
TBOPDP^b^	609	651	0.30	59 ± 2	0.12 ± 0.01
TBOEMC^b^	610	656	0.29	61 ± 3	0.17 ± 0.01
TBOPDP@CB[7]	608	646	0.46	65 ± 1	0.21 ± 0.01
TBOEMC@CB[7]	609	648	0.37	65 ± 4	0.19 ± 0.01
HSA-TBOPDP^b^	595	645	0.54	27 ± 8	0.020 ± 0.003
HSA-TBOEMC^b^	600	646	0.50	23 ± 7	0.022 ± 0.003
HSA-TBOPDP@CB[7]	595	643	0.46	27 ± 8	0.021 ± 0.003
HSA-TBOEMC@CB[7]	600	645	0.52	32 ± 2	0.026 ± 0.006

a Measurements in D_2_O buffer.

b Reference 19.

These experimental data are supported by computational calculations in which the interaction between CB[7] and TBO or its two derivatives, TBOPDP and TBOEMC, was studied through molecular docking (Figures 2 and S1). The results have shown that the TBO core fits inside the CB[7] cavity, consistent with the hydrophobic interactions with cationic dyes, reported in the literature.^5,20,22^ These interactions are driven by van der Waals forces and the release of high-energy water molecules from the CB[7] cavity, promoting a stable inclusion complex.^23^ This interaction is observed experimentally with the hypsochromic shift in the absorption and emission bands and a lengthening of the average fluorescence lifetimes. The binding energies of CB[7] with TBO, TBOPDP, and TBOEMC were −4.32, −4.30, and −4.11 kcal/mol, respectively. All of these complexes showed the formation of a hydrogen bond between a nitrogen atom of the amide group of TBOPDP and TBOEMC, acting as a donor, and the oxygen atom of the carbonyl group of CB[7] as an acceptor. These hydrogen bonds (N–H··O) had lengths of 2.2 and 2.1 Å, with a bond angle of 128.3°, stabilizing the complexes (Figure 2).

**Figure 2:**
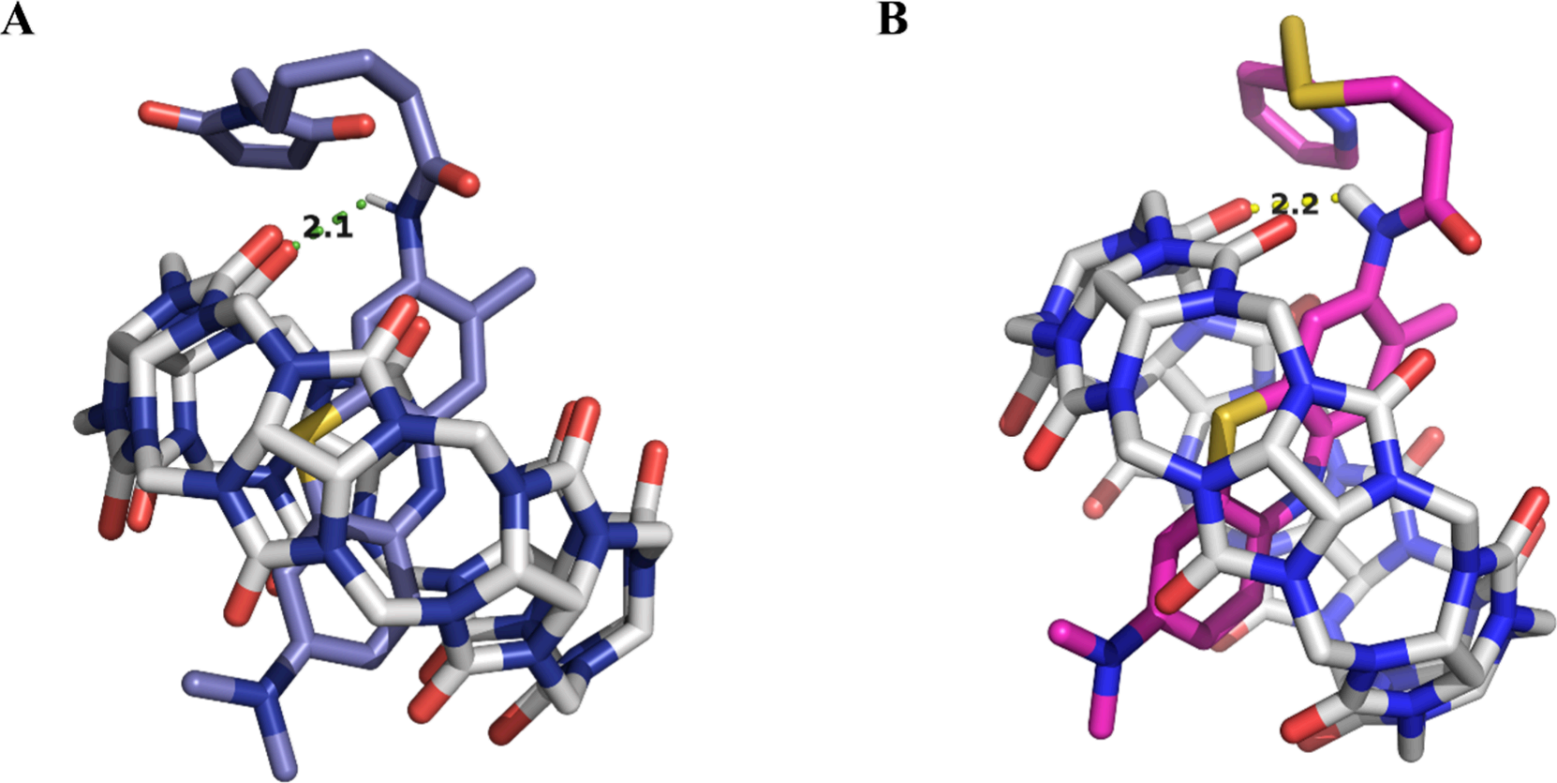
Molecular docking of (A) TBOPDP@CB[7] and (B) TBOEMC@CB[7]. Binding energies are −4.30 and −4.11 kcal mol^–1^, respectively.

The photophysical properties of the HSA-PS@CB[7] complexes were also evaluated. PS-HSA conjugation showed a slight hypsochromic shift in the absorption and emission bands (Table 1). In the case of TBOPDP, the average fluorescence lifetimes are identical for the free PS and its HSA conjugate upon complexation with CB[7] (Table 1). On the other hand, TBOEMC shows a lengthening of the average fluorescence lifetime. This difference could arise from the aliphatic chain length between the two TBO derivatives. Indeed, the shorter chain in TBOPDP could force a change in the PS conformation in its conjugated form upon complexation with CB[7].

### Binding Studies

The binding of TBO derivatives with CB[7] was analyzed through their binding isotherms, measured by fluorescence spectroscopy (see Figures S2 and S3 in the Supporting Information). The binding constant (*K*_b_) for the complexes TBOPDP@CB[7] and TBOEMC@CB[7] was determined according to eq 1 (see the Materials and Methods section). The analysis indicated that the inclusion complexes were formed at a 1:1 molar ratio, which is consistent with previous reports for TBO and other derivatives.^5,20^

The binding constants are reported in Table 2. Compared to the binding constant reported for TBO^5^ ((6.0 ± 1.0)× 10^6^ M^–1^), the *K*_b_ of its derivatives TBOPDP and TBOEMC decreased somewhat, although they remained fairly high compared to other macrocycles such as cyclodextrins.^24^

**Table 2:** Binding Constant, *K*_b_ (M^–1^), for TBO, TBOPDP, and TBOEMC with CB[7] Obtained from Fluorescence Data at 25 °C in 10 mM Phosphate Buffer at pH 7.0

compound	***K***_**b CB[7]**_ **/M** ^–1^
TBOPDP	(6.8 ± 2.4) × 10^5^
TBOEMC	(7.5 ± 3.0) × 10^4^

### Singlet Oxygen Measurements

The singlet oxygen quantum yield (Φ_Δ_) of TBO derivatives with HSA and CB[7] is reported in Table 1, and the actual measurements are depicted in Figures S4–S6 in the Supporting Information. The supramolecular complexes of TBO derivatives with CB[7] showed a slight increase in singlet oxygen generation, due to the restricted mobility mentioned above, which improved photophysical properties.^25^ This result is contrasting with our previous work, where we showed a decrease in singlet oxygen quantum yield for TBO derivatives conjugated with HSA, likely due to limited oxygen accessibility in the Cys34 pocket, as well as interaction with the neighboring reactive microenvironment such as Histidine-39 and Tyrosine-84.^19^

In the case of HSA conjugates with CB[7], only complex HSA-TBOEMC@CB[7] exhibited a slight improvement in the singlet oxygen quantum yield, from 0.022 to 0.026, compared to HSA-TBOEMC alone. This enhancement could be attributed to the longer aliphatic chain of TBOEMC, which improves its accessibility to oxygen protruding out of the CB[7] cavity. Overall, the singlet oxygen quantum yield for CB[7] complexes was comparable to, or even higher than, that of free TBO^19^ with a value of 0.14, while HSA conjugates exhibited lower yields (Table 1).

### Cell Uptake Studies

Despite the variation in singlet oxygen production, the PDT efficacy of these systems was evaluated based on their in vitro studies regarding incorporation and phototoxicity. To assess the impact of each derivate and delivery system on cellular uptake, we quantified the total PS content (picomol/10^6^ cells) after cellular lysis, using standard curves for each PS (Figure 3).

**Figure 3:**
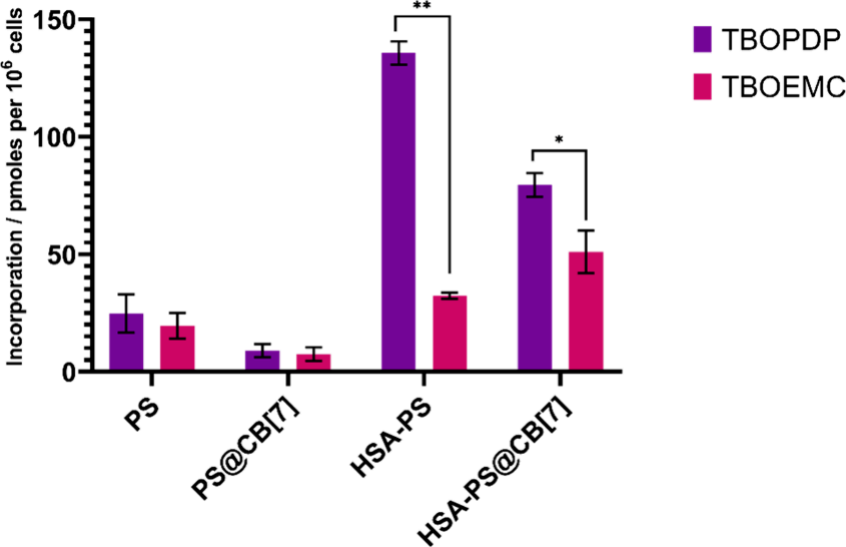
Cell uptake after 90 min of incubation of PS with different systems. Concentrations of the systems were 3 μm PS, 3 μm HSA-PS, and 50 μm CB[7]. Statistical significance: *p* < 0.05 (*), *p* < 0.01 (**).

The results indicate that complexation with CB[7] lowers cell uptake, but the difference was not statistically significant compared with the free compounds. Specifically, TBOPDP exhibited an incorporation of 24.71 ± 8.13 picomol/10^6^ cells, while the TBOPDP@CB[7] complex showed an uptake of 8.95 ± 2.74 picomol/10^6^ cells. A similar trend was observed for TBOEMC, displaying an incorporation of 19.55 ± 5.46 picomol/10^6^ cells, whereas TBOEMC@CB[7] showed an uptake of 7.45 ± 2.94 picomol/10^6^ cells. While the cellular uptake of the CB[7] complex is not improved, this does not imply that complexation is without benefits, as previous studies and the current work have shown important enhancements in photophysical properties.^5^

On the other hand, the use of HSA as DDS led to significantly higher cellular incorporation. The conjugates HSA-TBOPDP and HSA-TBOEMC showed an increase in cell uptake compared with both the free derivatives and inclusion complexes with CB[7]. This increase can be attributed to the high affinity of HSA for cancer cells, mediated by receptors like gp60, the neonatal Fc receptor (FcRn), and SPARC (secreted protein, acidic and rich in cysteine), which are known to enhance cellular uptake via endocytosis.^26^ These findings reaffirm the extraordinary potential of HSA conjugations to improve PS delivery in cancer.

The HSA-TBOPDP conjugate exhibited a cellular incorporation of 135.65 ± 5.01 picomol/10^6^ cells, approximately five times higher than that of free TBOPDP and 15 times higher than for the TBOPDP@CB[7] complex. In comparison, HSA-TBOEMC showed an incorporation of 32.33 ± 1.34 picomol/10^6^ cells, which, although lower than that of HSA-TBOPDP, was still significantly greater than that of TBOEMC or the TBOEMC@CB[7] complex. This difference in uptake behavior between HSA-TBOPDP and HSA-TBOEMC suggests that the type of bond formed with the Cys34 residue of HSA plays a critical role. HSA-TBOPDP forms a disulfide bond with Cys34, whereas HSA-TBOEMC forms a thioether bond. Disulfide bonds are more labile and susceptible to intracellular reduction by reducing agents such as glutathione, which is overexpressed in cancer cells.^27^ This reduction likely facilitates the release of the PS from the HSA-TBOPDP conjugate, enhancing its intracellular retention.^28^ In contrast, the thioether bond in HSA-TBOEMC remains more stable under physiological conditions, though it also may still undergo thiol exchange or hydrolysis via retro-Michael reactions.^29^

This bond stability becomes relevant when HSA is incorporated into cells via the neonatal Fc receptor (FcRn), a pathway associated with endocytic recycling. Through this mechanism, HSA can be relocalized to the extracellular space, facilitating both recycling and degradation.^30^ Based on this process, it is likely that the HSA-TBOEMC conjugate, which forms a more stable thioether bond, remains intact during cellular uptake. However, due to the stability of this bond, a portion of the conjugate may also be expelled during recycling, leading to reduced intracellular PS accumulation.

In contrast, the HSA-TBOPDP conjugate, linked via a more labile disulfide bond, could be reduced by glutathione or other redox systems, facilitating PS release within the cell. Consequently, when HSA undergoes recycling, the PS is not carried back to the extracellular space, unlike in the case of TBOEMC. These differences in bond stability between the two conjugates can therefore be exploited to control intracellular PS accumulation.

Furthermore, the addition of CB[7] to the conjugates, that is, the HSA-TBOPDP@CB[7] and HSA-TBOEMC@CB[7] complexes, led to statistically significant differences compared to their CB[7] free counterparts, with cellular incorporations of 79.50 ± 5.03 and 51.03 ± 9.08 picomol/10^6^ cells, respectively. The underlying mechanism for this effect remains unclear. As observed with other TBO derivatives, CB[7] alone does not enhance PS uptake.^31^ However, once the PS is conjugated to HSA, CB[7] appears to modulate uptake differently depending on the conjugate.

In the case of HSA-TBOPDP, a significant decrease in cellular uptake was observed; meanwhile, for the case of HSA-TBOEMC, the effect was the opposite. For the HSA-TBOPDP conjugate, it is plausible that CB[7] binding near the disulfide bond inhibits its reduction, leading to an increase in PS expulsion during HSA recycling. On the contrary, for the TBOEMC adduct, the longer linker chain may favor the reverse thia-Michael reaction or exchange with biological thiols such as glutathione.

These results highlight the crucial role of HSA in enhancing PS incorporation and CB[7] in protecting PS and boosting its photoactivity.

### Cell Phototoxicity

First, we explored the phototoxicity of TBO, TBOPDP, and TBOEMC at different concentrations between 0.4 and 40 μM, which showed that concentrations higher than 0.4 μM showed extensive phototoxicity and dark cytotoxicity (see Figure S7 in the Supporting Information). Thus, all of the experiments with the delivery systems were carried out using a concentration of 0.4 μM. We examined the phototoxicity of the different complexes and conjugates. Dark experiments confirmed that none of the evaluated systems exhibited cytotoxicity under the conditions evaluated (see Figure S8 in the Supporting Information). In contrast, upon illumination, HeLa cells treated with TBOPDP and TBOEMC using different DDS displayed notable phototoxic effects. Control experiments with HSA and CB[7] showed no cytotoxicity, consistent with a previous work^32^ (see Figure S9 in the Supporting Information).

For TBOPDP (Figure 4a), no statistically significant differences in phototoxicity were observed among the different DDSs, with cell viability remaining around 70% across all systems. Similar results were observed for TBOEMC (Figure 4b), where the phototoxicity remained constant regardless of the DDS employed. Although the singlet oxygen quantum yields of the HSA bioconjugates are lower compared to those with CB[7], the increased cellular uptake compensates for this difference, playing a crucial role in phototoxocity.

**Figure 4:**
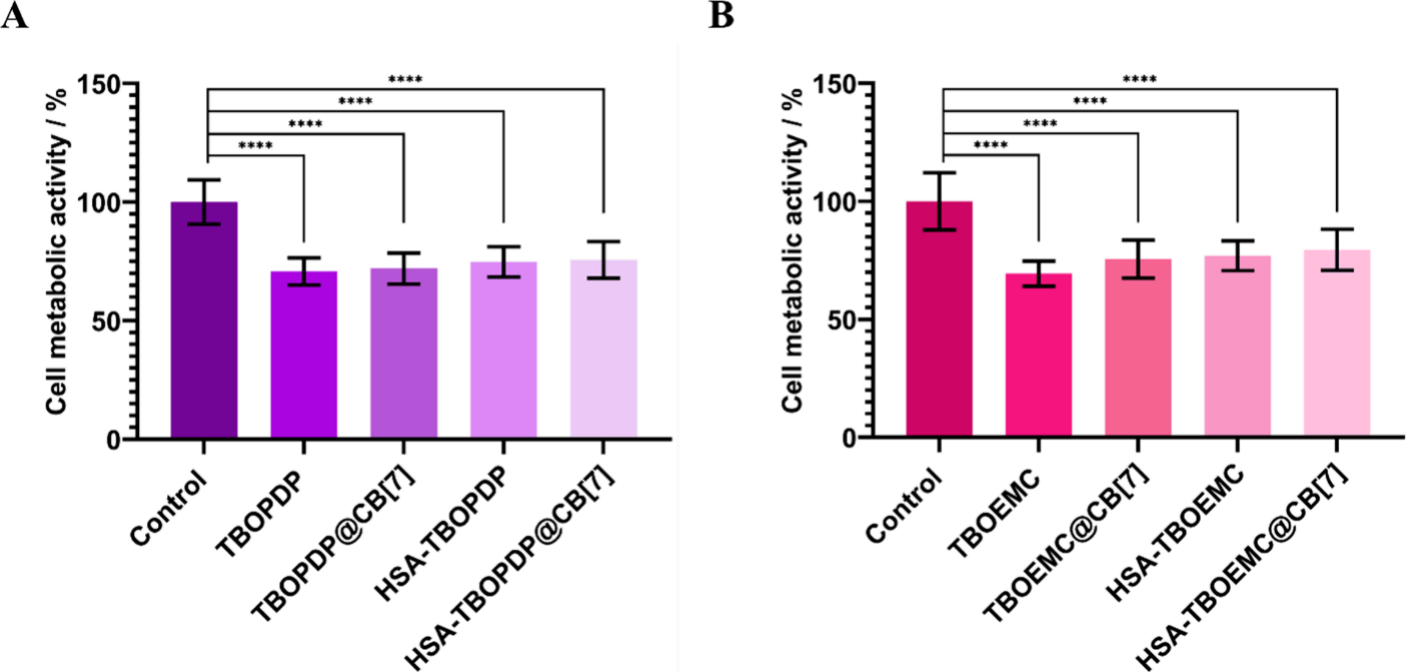
Comparison of the phototoxicity of different systems in HeLa cell metabolic activity under light conditions. PS corresponds to (A) TBOPDP and (B) TBOEMC. Concentrations of the systems were 3 μM PS, 3 μM HSA-PS, and 50 μM CB[7]. Incubation and irradiation were for 90 min with 630 nm LEDs. Statistical significance of *p* < 0.0001 (****).

While the similarities among DDS may suggest comparable phototoxic effects, a more comprehensive analysis considering key parameters, such as singlet oxygen quantum yield and cellular uptake, reveals distinct differences. Table S1 summarizes the data on cell uptake and cell viability along with the relationship between these parameters and singlet oxygen generation. Upon trend examination, we found out that there is a linear correlation between singlet oxygen generation and phototoxic efficiency once cell viability is corrected by the cellular uptake of the different systems (see Figure S10 in the Supporting Information). It is interesting to note that in the literature, it is difficult to find a correlation between singlet oxygen quantum yields of PS and their phototoxicity. Normally it is assumed that different subcellular localizations or changes in the mechanism of generation of ROS inside cells explain this lack of correlation. However, as observed herein, the critical factor might be to incorporate cell uptake in these measurements, together with the quantification of singlet oxygen generation by direct methods.

This comparison highlights the multifactorial nature of the phototoxic efficiency. When HSA is used as a DDS, it reduces the singlet oxygen quantum yield, as previously discussed. However, its high cellular uptake compensates for this limitation, enhancing the phototoxicity. Conversely, CB[7] nanocapsules exhibit similar or lower cellular uptake, but this is compensated for with a superior singlet oxygen generation capability, ultimately boosting phototoxic efficiency. The balance between these two opposite effects explains why the phototoxicity remained constant even when large differences in singlet oxygen generation and cell uptake were observed. These findings highlight the importance of considering all contributing factors in the design and evaluation of future DDSs for optimized PDT performance.

## Conclusions

The impact of DDSs on the PDT application of TBO derivatives, specifically TBOPDP and TBOEMC, is crucial for optimizing their efficacy. In this work, we showed that HSA bioconjugates with TBO derivatives have increased cellular uptake in tumoral cells. The TBO derivatives formed stable inclusion complexes with CB[7], which also form when TBO derivatives are conjugated with HSA. The combination of both systems influences the overall properties of the bioconjugates. On the one hand, HSA decreases fluorescence emission and singlet oxygen quantum yields, and on the other hand, CB[7] restores the fluorescence emission and singlet oxygen generation by protecting the PS like a nanocapsule. Addition of CB[7] also shows a dramatic effect on the cellular uptake, increasing incorporation for TBOEMC and decreasing incorporation for TBOPDP. We suggest that this is related to the lability of the covalent disulfide and thia-Michael adduct in the intracellular media. Moreover, phototoxicity results in vitro highlight the relationship among cellular viability, cell uptake, and singlet oxygen generation in determining the phototoxic efficiency, emphasizing the importance of a more rounded approach to PDT optimization. Overall, these bioconjugates and supramolecular complexes show good potential for applications in PDT.

## Materials and Methods

TBO derivatives (TBOPDP and TBOEMC) were synthesized from purified TBO,^8^ as previously reported.^19^ Rose Bengal (RB), human serum albumin (HSA), cucurbit[7]­uril (CB[7]), bis­(cyclopentadienyl)­cobalt­(III) hexafluorophosphate (Cob^+^), 4,5-(dimethyl-2-thiazole)-2,5-diphenil-2H-tetrazolium bromide (MTT), and solvents were used as received from Sigma-Aldrich. HSA conjugates were prepared as previously reported, and their characterization was confirmed by MALDI-TOF mass spectrometry (Supporting Information Figure S11). Dulbecco's modified Eagle’s medium (DMEM) and fetal bovine serum (FBS) were obtained from Sigma and HyClone. Trypsin, penicillin, streptomycin, and amphotericin b were obtained from Gibco. All reagents were of the highest purity available.

### Stock Solutions

TBO and its derivative working solutions were prepared at a concentration of 1 mM in methanol, and the concentration was assessed by UV–vis spectroscopy using their molar extinction coefficients.^8^ Phosphate buffer (10 mM, pH 7) was prepared from disodium hydrogen phosphate (Na_2_HPO_4_) and sodium dihydrogen phosphate (NaH_2_PO_4_). CB[7] working solution was prepared from an aqueous 1 mM stock solution of CB[7], and the concentration was determined using Cob^+^ titration, as reported in the literature.^33^ For in vitro experiments, the final concentrations of 3 μM for TBO, TBOPDP, TBOEMC, HSA-TBOPDP, and HSA-TBOEMC and 50 μM for CB[7] were prepared in unsupplemented DMEM media without phenol red. The amount of methanol in the solutions was less than 0.1% in all cases.

### Absorption and Fluorescence Measurements

Absorption spectra of TBO derivatives and the different complexes with HSA and CB[7] were collected in a Hewlett-Packard 8453 UV–Vis spectrophotometer using cuvettes with 1 cm path length. Fluorescence emission spectra were recorded using a PerkinElmer LS55 fluorimeter, with a slit width equivalent to a bandwidth of 2.5 nm, and the samples were excited at the absorption maxima.

### Fluorescence Lifetimes

A LifeSpecII fluorescence lifetime spectrometer (Edinburgh Instruments) was used to determine the fluorescence lifetimes using the TCSPC technique. Samples were excited with a laser diode of 638 nm for samples in phosphate buffer at pH 7.0 (10 mM). The number of counts was set according to the previously reported procedure.^19^ Lifetimes were obtained from the fluorescence decay analysis using the reconvolution fit from the F980 software. The goodness of the fit was judged from the residual distribution around zero and χ2 values between 0.9 and 1.2.

### Binding Affinities for CB[7]

The binding constant of the derivatives TBOPDP and TBOEMC with CB[7] was determined using a binding isotherm. Titrations were performed by increasing the concentration of the CB[7] from 0 to 100 μM while keeping the concentration of PS (TBOPDP or TBOEMC) constant at 2 μM. All measurements were performed in phosphate buffer at pH 7.0 (10 mM). Data analysis was carried out using the changes in the fluorescence intensity (I) using a nonlinear adjustment (eq 1) to determine the binding constant (*K*_b_).

$$I=I_{\mathrm{PS}}[\mathrm{PS}]+I_{\mathrm{PS}@\mathrm{CB}7}-I_{\mathrm{PS}}(\frac{1}{2}\{([\mathrm{PS}]+[\mathrm{CB}7]+\frac{1}{K_{b}})-\sqrt{\left[\mathrm{PS}]+[\mathrm{CB}7]++\frac{1}{K_{b}}-4[\mathrm{CB}7][\mathrm{PS}\right]}\})$$ 1

eq 1. Equation used to determine the binding constant.

where *I*_PS_ and *I*_PS_@CB7 are the fluorescence intensity of the derivate and complex with CB[7], respectively. [PS] and [CB7] are the concentrations of the photosensitizer and CB[7] during the experiment.^34^

### Singlet Oxygen Measurements

Direct detection of singlet oxygen (^1^O_2_) generated by the different systems was performed using a customized system of time-resolved near-infrared (NIR) phosphorescence at 1270 nm.^35^ The samples were excited at 532 nm using a diode-pumped pulsed Nd:YAG laser (FTSS355-Q3, CryLaS Laser Systems GmbH, Berlin, Germany) working at a 1 kHz repetition rate. The detection of ^1^O_2_ phosphorescence was measured with a Hamamatsu NIR detector (H10330A-45, operating at −908 V) coupled to a grating monochromator (CM110–1/8m, Spectral Products, Digikröm). A rugate notch filter (1064 nm, Edmund Optics, York, UK) and a long-pass filter (1150 nm, FEL1150, ThorLabs, Newton, NJ) were placed at the entrance port of the monochromator to remove any residual component of the laser fundamental emission in the near-infrared region. Photon counting was reached with a multichannel scaling card (TimeHarp 260-NANO, PicoQuant GmbH, Germany). The phosphorescence signals were collected in air-equilibrated deuterated phosphate buffer for 500 s with a 256 ns resolution. All of the measurements were performed in triplicate.

The kinetics were analyzed using the expected rise and decay biexponential model of ^1^O_2_, which is described by eq 2. The phosphorescence signal (*S*_(*t*)__)_) is a function of the intensity of the phosphorescence of ^1^O_2_ at time zero (*S*_0_); the ^1^O_2_ lifetime (τ_Δ_), the PS triplet lifetime (τ_T_), and the term *Y*_0_ are added to account for the baseline in the real measurement.^36^ The fitting of the signals was carried out using GraphPad Prism 8.0 software (GraphPad Software, Inc., La Jolla, CA) with τ_Δ_, τ_T_, *S*_0_, and *Y*_0_ as free parameters. The quality of the fitting was assessed from the residual plots.

$$S_{\left(t\right)}=S_{0}\frac{\tau_{\Delta }}{\left(\tau_{\Delta }-\tau_{T}\right)}(e^{\left(-t/\tau_{\Delta }\right)}-e^{\left(-t/\tau_{T}\right)})+Y_{0}$$ 2

eq 2. Biexponential model for the formation and decay of ^1^O_2_.

Singlet oxygen quantum yield (Φ_Δ_) was determined using eq 3. Rose bengal (RB, Φ_Δ_^R^ = 0.76) was used as the reference in deuterated phosphate buffer.^37^ The determination of Φ_Δ_ was carried out using different concentrations of PS or HSA-PS in the presence of excess of CB[7], and the maximum intensity of the phosphorescence of singlet oxygen (*S*_0_) was plotted against the intensity of light absorbed at the excitation wavelength by the sample (1–10^–Abs 532 nm^)^S^ and the reference photosensitizer RB (1–10^–Abs 532 nm^)^R^. The resulting plots were fitted with a linear equation (see the Supporting Information, Figure S6), and the Φ_Δ_ values were determined from the slopes (*S*_0_/(1–10^–Abs532 nm^).^37^ The Φ_Δ_ values of the supramolecular complexes (TBOPDP@CB[7] and TBOEMC@CB[7]) were determined by comparing the slopes of *S*_0_ for the 1270 nm signals of optically matched samples (see the Supporting Information, Figure S9). Experiments were done in triplicate.

$${\Phi_{\Delta }}^{S}={\Phi_{\Delta }}^{R}\frac{{S_{0}}^{S}}{{S_{0}}^{R}}\frac{(1-10^{-\mathrm{Abs}\;532\;\mathrm{nm}}{)}^{R}}{(1-10^{-\mathrm{Abs}\;532\;\mathrm{nm}}{)}^{S}}$$ 3

eq 3. Quantum yield of the ^1^O_2_ generation equation.

### Docking Studies

The structural optimization of the derivatives TBOPDP and TBOEMC was obtained using Spartan 10, as was described previously.^20^ The main interaction of the complexes of derivatives with CB[7] was evaluated by molecular docking studies using AutoDock 4.02 and AutoDock Tools.^38^ Grid maps were centered on the cavity of CB[7] to generate the supramolecular complexes. The volume for the grid maps was generated using autogrid4, with a grid-point spacing of 0.375 Å. Lamarckian genetic algorithm docking was performed using the following parameters: a population size of 150 individuals, 2.5 × 10^6^ energy evaluations, a maximum number of 27,000 generations, a mutation rate of 0.02, and a crossover rate of 0.80. The most favorable complexes were chosen with the lowest energy score and high numbers of conformations. PyMol was employed for structural visualization, data analysis, and image generation.^39^

### Cell Culture Studies

In vitro studies of TBOPDP and TBOEMC (3 μM) with delivery systems such as HSA (6–7 μM) and CB[7] (50 μM) were assessed in HeLa cells. HeLa cells were cultured in Dulbecco’s modified Eagle’s medium (DMEM) with phenol red and supplemented with 10% fetal bovine serum (FBS), antibiotic, and antimycotic solution (100 μg/mL streptomycin, 100 U/mL penicillin, and 0.25 μg/mL amphotericin B). This medium will be referred to as MC10% hereafter. Cells were incubated at 37 °C, 5% atmosphere of CO_2_, and 100% humidity. HeLa cells were subcultured using standard trypsin protocols. Cell counting was performed using a Carl Zeiss Axiover 25, trypan blue, and a standard Neubauer chamber. The different systems comprising PSs and DDSs and their complexes were incorporated by using DMEM without phenol red and FBS.

### Cell Uptake

HeLa cells were seeded at a density of 2.0 × 10^5^ cells/well into a 6-well plate and cultured overnight in supplemented DMEM until cells reached a 70–80% confluence. The medium was removed, and the cells were washed three times with DMEM without phenol red. The different systems were then added at the specified concentrations (see the previous section) and incubated for 90 min. After the incubation period, the cells were washed three times with HBSS (Hank’s Balanced Salt Solution), then trypsinized, and centrifugated. The supernatant was discarded, and the pellet was resuspended in 2 mL of HBSS before being centrifuged again. The final pellet was resuspended in 500 μL of 2% SDS and incubated overnight at 37 °C. The concentration of the different incorporated systems (picomol/10^6^ cells) was determined using a standard fluorescence calibration curve (intensity vs PS concentration).

### Dark Cytotoxicity

HeLa cells were cultured to 80% confluence, plated into a 96-well plate at the seeding density of 1.5 × 10^5^ cells/well, and incubated overnight. The different systems were prepared in DMEM supplemented with 3% FBS, antibiotic, and antimycotic solution (MC3%). Cell viability was measured after incubation for 24 h in dark conditions using the cell viability assay.

### Phototoxicity Studies

Herein, the different systems (PS, PS@CB[7], HSA-PS, HSA-PS@CB[7]) were prepared using DMEM without phenol red and FBS. The concentration was set at 3 μM for the PSs and their HSA conjugates, while the HSA-PS@CB[7] complexes were prepared by adding 50 μM CB[7] to these systems. The controls corresponding to the delivery systems alone were prepared at a concentration of 6–7 μM HSA and 50 μM CB[7] (see the Supporting Information, Figure S9).

HeLa cells were seeded into a 96-well plate at a density of 1.5 × 10^5^ cells/well and cultured overnight. The medium was removed by aspiration and replaced with 100 μL of the different systems, followed by 90 min of incubation. After removal of the different systems via aspiration, the cells were rinsed twice with PBS and fresh DMEM (without phenol red) was added. The plate was then irradiated for 90 min using a Luzchem LEDL16 photoreactor (630 nm, light intensity 2.93 mW/cm^2^, and 37 °C). Finally, the DMEM was replaced with MC10% and incubated for 24 h for the viability assay. Dark controls were prepared under the same conditions but without irradiation. Statistical data analysis was performed using GraphPad Prism 8.0 software (GraphPad Software, Inc., La Jolla, CA), using the one-way ANOVA test to determine statistical significance. Statistical significance was considered with a confidence interval of *p* < 0.05.

## Supplementary Material



## Notes

Molecular docking, time-resolved phosphorescence signals, binding isotherm, cytotoxicity controls (PDF)

## #

Department of Chemistry, University of Calgary, Calgary T2N 1N4, Alberta, Canada

## Abbreviations

PDT: photodynamic therapy
TBO: toluidine blue O
HSA: human serum albumin
CB­[7]: cucurbit­[7]­uril
PS: photoactive drug
ROS: reactive oxygen species
DDS: drug delivery systems
RB: rose bengal
DMEM: Dulbecco's modified Eagle’s medium
^1^O_2_: singlet oxygen
