# Supplementary material for: Bioconjugates of Toluidine Blue Derivatives with Human Serum Albumin and Their Complexes with Cucurbit[7]uril as Drug Delivery Vehicles for Photodynamic Therapy
Source: ACS Omega. 2025 Oct 27;10(44):52333–42. doi: 10.1021/acsomega.5c03731 (PMC12612866; doi:10.1021/acsomega.5c03731)
Supplement: Supplementary file 1 [file ao5c03731_si_001.pdf]

Supplementary Material

Bioconjugates of toluidine blue derivatives with human serum albumin and their complexes with cucurbit[7]uril as drug delivery vehicles for photodynamic therapy

Nory Mariño-Ocampo [a#], José Robinson-Duggon [b,c], Daniel Zúñiga-Núñez [a], Daniel Guerra Díaz [d], Belinda Heyne [e] and Denis Fuentealba [a*]*

[a] Laboratorio de Química Supramolecular y Fotobiología, Departamento de Química Física, Escuela de Química, Facultad de Química y de Farmacia, Pontificia Universidad Católica de Chile, Vicuña Mackenna 4860, Código postal 7820436, Santiago.

[b] Universidad de Panamá, Facultad de Ciencias Naturales, Exactas y Tecnología, Departamento de Bioquímica, Panamá 0824, Código postal 0824-00110, República de Panamá.

[c] Sistema Nacional de Investigación (SNI), Secretaría Nacional de Ciencia, Tecnología e Innovación (SENACYT), Panamá, República de Panamá.

[d] Departamento de Química Orgánica, Facultad de Ciencias, Universidad de Chile, Las Palmeras 3425, Código postal 7800003, Santiago, Chile.

[e] Department of Chemistry, University of Calgary, 2500 University Drive NW, T2N 1N4, Calgary, AB, Canada.

[#]Present address: Department of Chemistry, University of Calgary, 2500 University Drive NW, T2N 1N4, Calgary, AB, Canada.

Corresponding authors: Nory Mariño-Ocampo (njmarino@uc.cl), Denis Fuentealba (dlfuente@uc.cl)

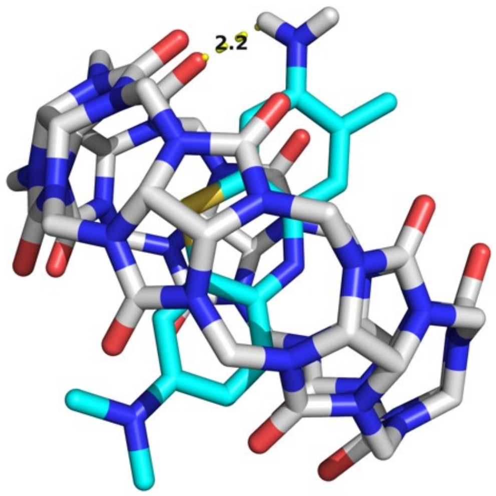

Figure S1. Molecular docking of TBO@CB[7]. Binding energy -4.32 kcal mol^{-1}.

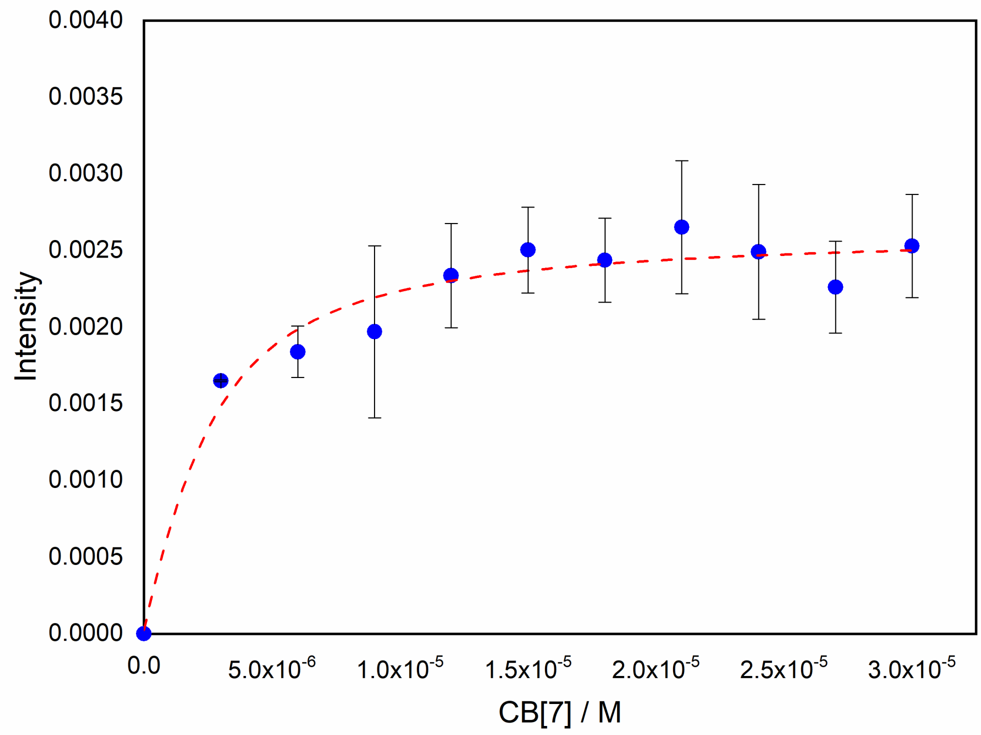

Figure S2. Binding Isotherm based on the fluorescence intensity of TBOPDP (2 μM) in 10 mM phosphate buffer pH 7.0 in presence of different concentration of CB[7] (0-30 μM) excited at 610 nm and emission collected at 646 nm. Data were fitted using numerical analysis.

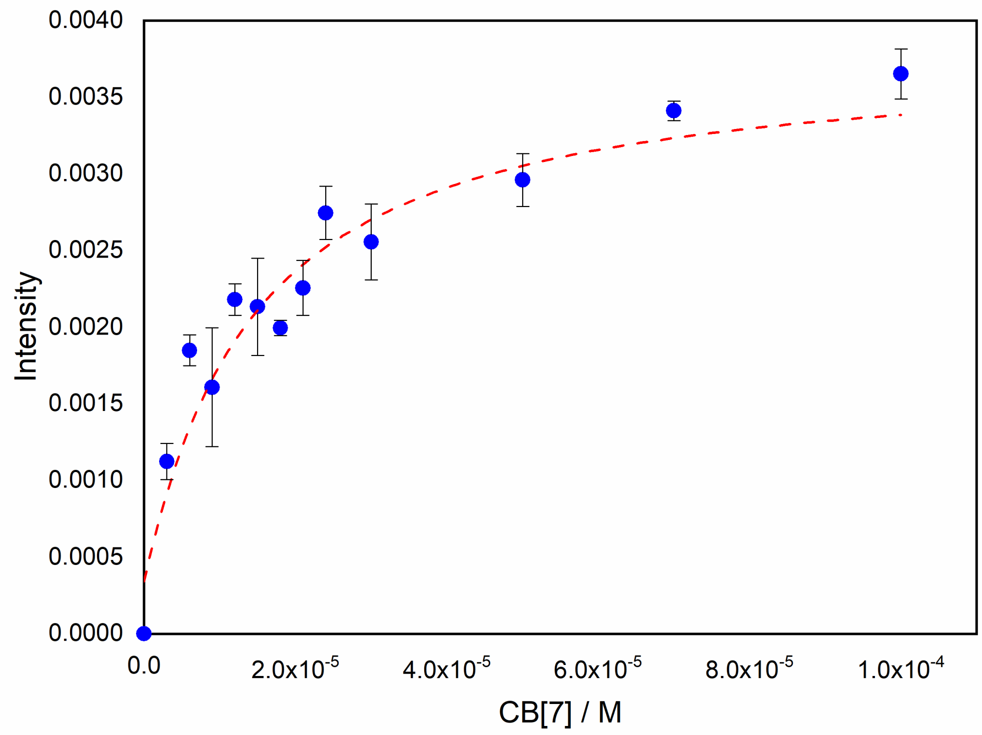

Figure S3. Binding Isotherm based on the fluorescence intensity of TBOEMC (2 μM). in 10 mM phosphate buffer pH 7.0 in presence of different concentration of CB[7] (0-100 μM) excited at 610 nm and emission collected at 648 nm. Data were fitted using numerical analysis.

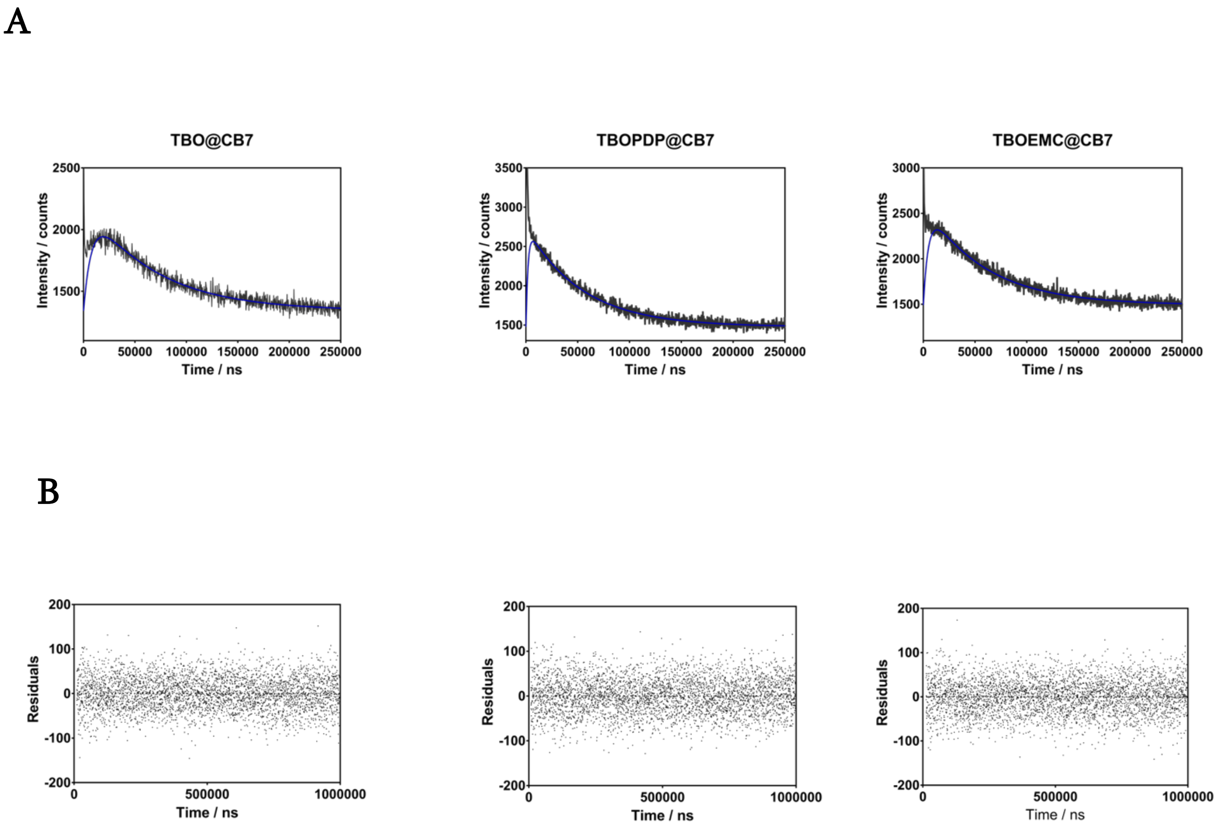

Figure S4. Time-resolved phosphorescence signals obtained in 10 mM phosphate buffer pH 7.0 at 1270 nm for: (A) TBO (left, black), TBOPDP (center, black), and TBOEMC (right, Black) and the corresponding fittings (Blue). (B) Residuals plots obtained from the fits.

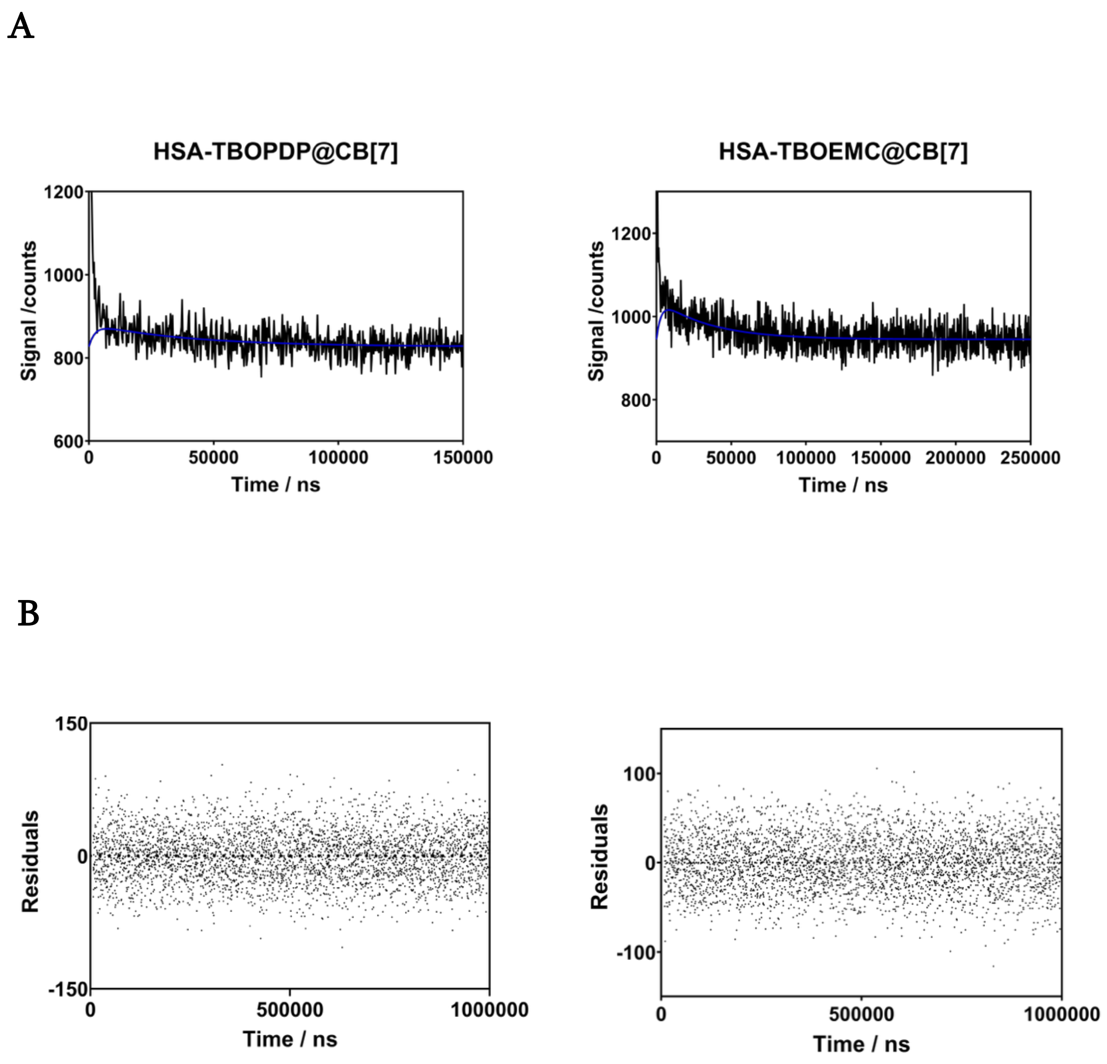

Figure S5. Time-resolved phosphorescence signals obtained in 10 mM phosphate buffer pH 7.0 at 1270 nm for biosupramolecular complexes: (A) HSA-TBOPDP@CB[7] (left, black), and HSA-TBOEMC@CB[7] (right, Black) and the corresponding fittings (Blue). (B) Residuals plots obtained from the fits.

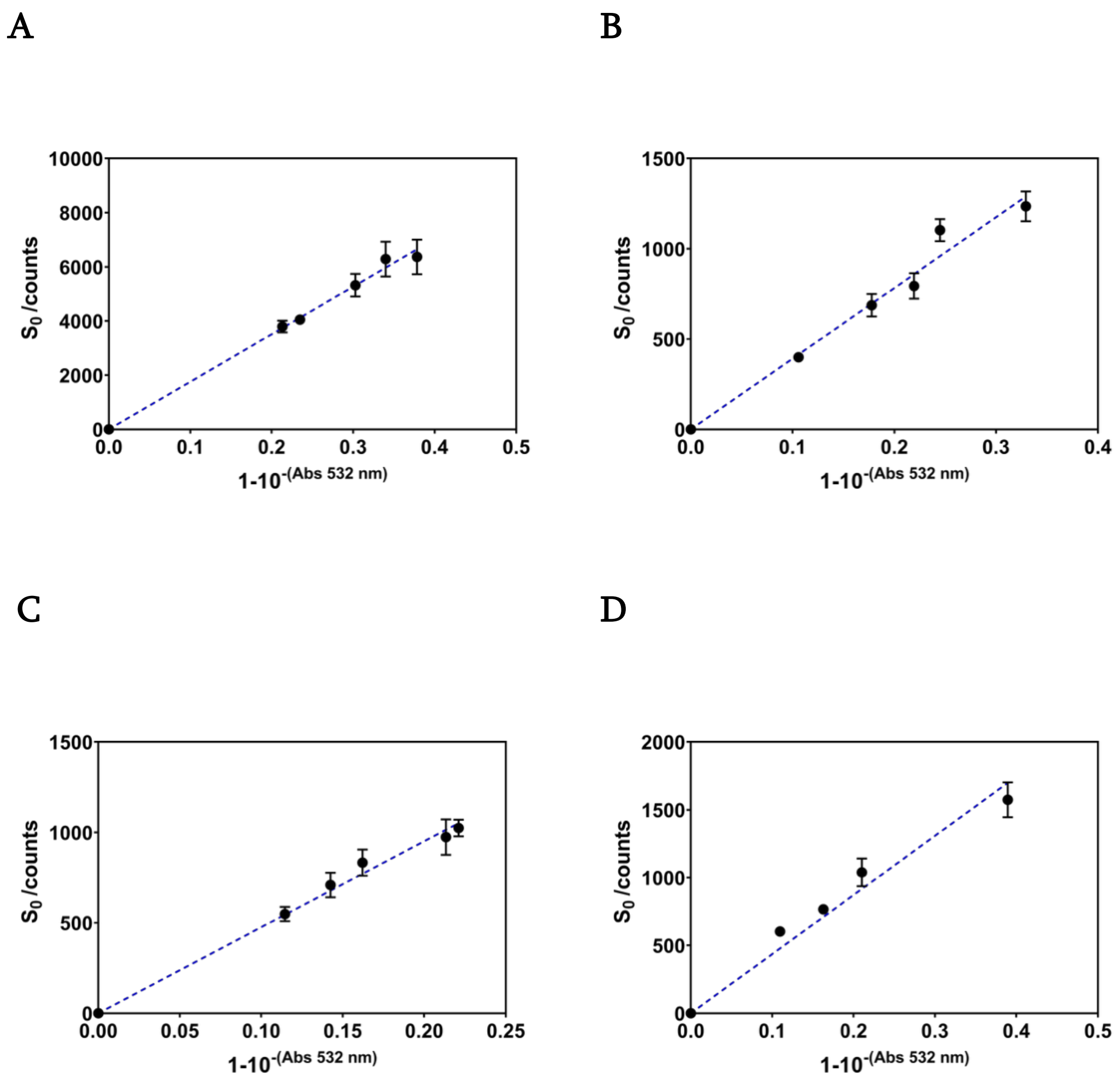

Figure S6. Intensity of time-resolved $^{1}O_2$ phosphorescence signals obtained in 10 mM phosphate buffer pH 7.0 at 1270 nm for: (A) RB (slope= 17572 ± 288.5, (B) TBO@CB[7] (slope= 3917 ± 147.6), (C) TBOPDP@CB[7] (slope= 4759 ± 96.8), and (D) TBOEMC@CB[7] (slope= 4363 ± 228.9), at different concentrations.

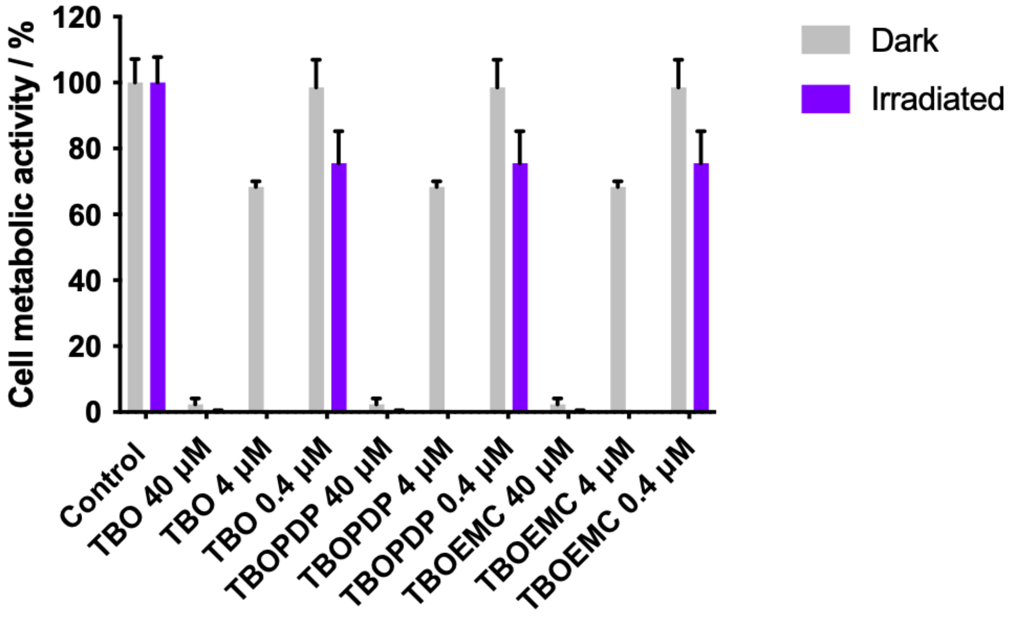

Figure S7. Comparison of the cytotoxicity of different systems in HeLa cells metabolic activity under dark (gray bars) and irradiated (purple bars) conditions. Incubation and irradiation for 90 minutes with 630 nm LEDs.

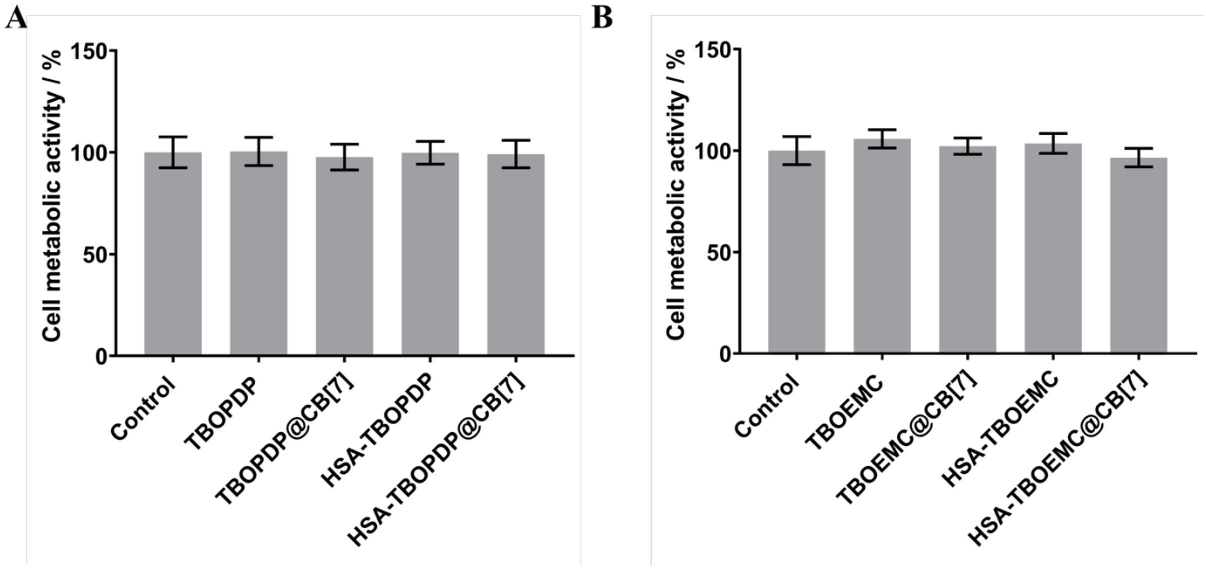

Figure S8. Comparison of the cytotoxicity of different systems in HeLa cells metabolic activity under dark conditions. PS correspond to (a) TBOPDP and (b) TBOEMC. Concentration of the systems were 3 µm PS, 3 µm HSA-PS, and 50 µm CB[7]. Incubation for 90 minutes. Statistical significance of $p < 0.05$ (*).

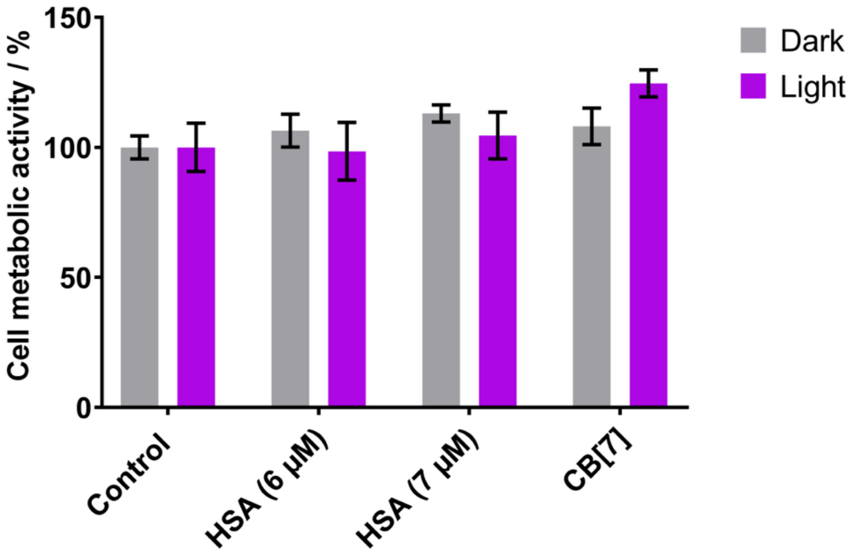

Figure S9. Comparison of the cytotoxicity of different systems in HeLa cells metabolic activity under dark and light conditions. Concentration of of the systems were 3 μm PS, 3 μm HSA-PS, and 50 μm CB[7]. Incubation for 90 minutes. Statistical significance of $p < 0.05$ (*).

Table S1. Summary of the performance efficiency of each system in cell studies.

	Cell uptake	Cell viability	(100-cell viability)/cell uptake	ϕ_Δ
TBOPDP	24.71±8.13	70.79±5.74	1.2	0.12
TBOPDP@CB[7]	8.95±2.74	72.03±6.52	3.1	0.21
HSA-TBOPDP	135.64±5.01	74.84±6.39	0.2	0.02
HSA-TBOPDP@CB[7]	79.50±5.03	75.66±7.74	0.3	0.02
TBOEMC	19.55±5.46	69.36±5.35	1.6	0.17
TBOEMC@CB[7]	7.45±2.94	75.32±8.02	3.3	0.19
HSA-TBOEMC	32.32±1.34	76.96±6.32	0.7	0.02
HSA-TBOEMC@CB[7]	51.03±9.08	79.41±8.70	0.4	0.03

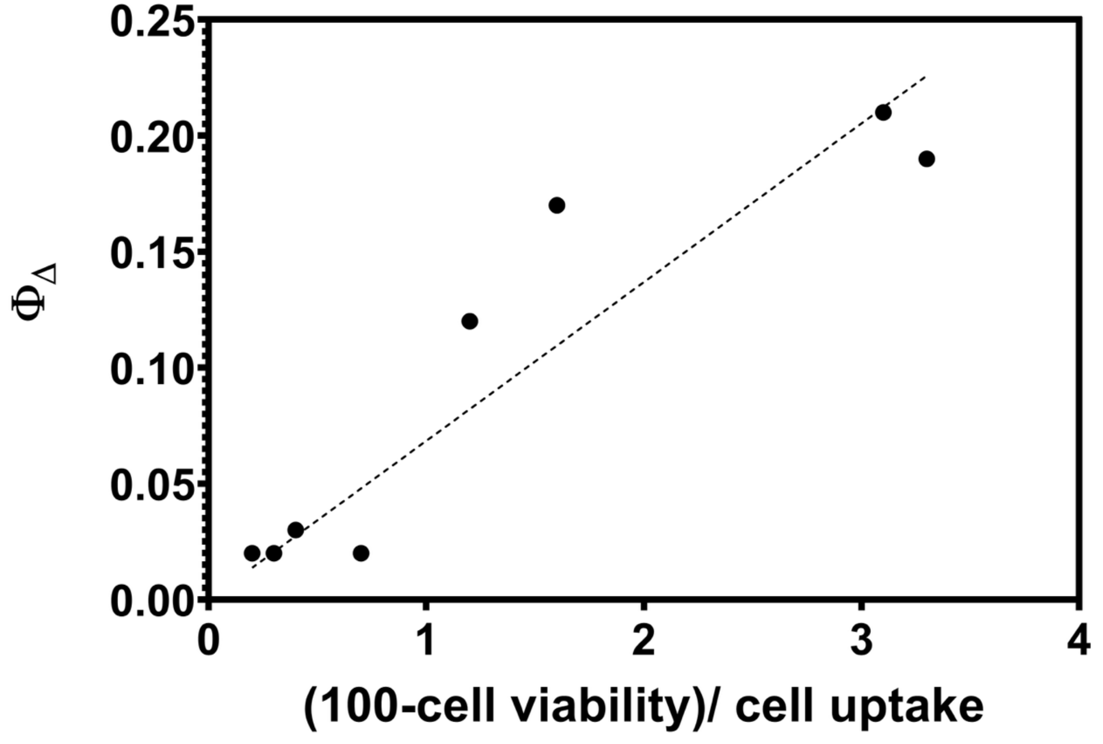

Figure S10. Phototoxicity efficiency vs singlet oxygen quantum yield. R^2 = 0.9394.

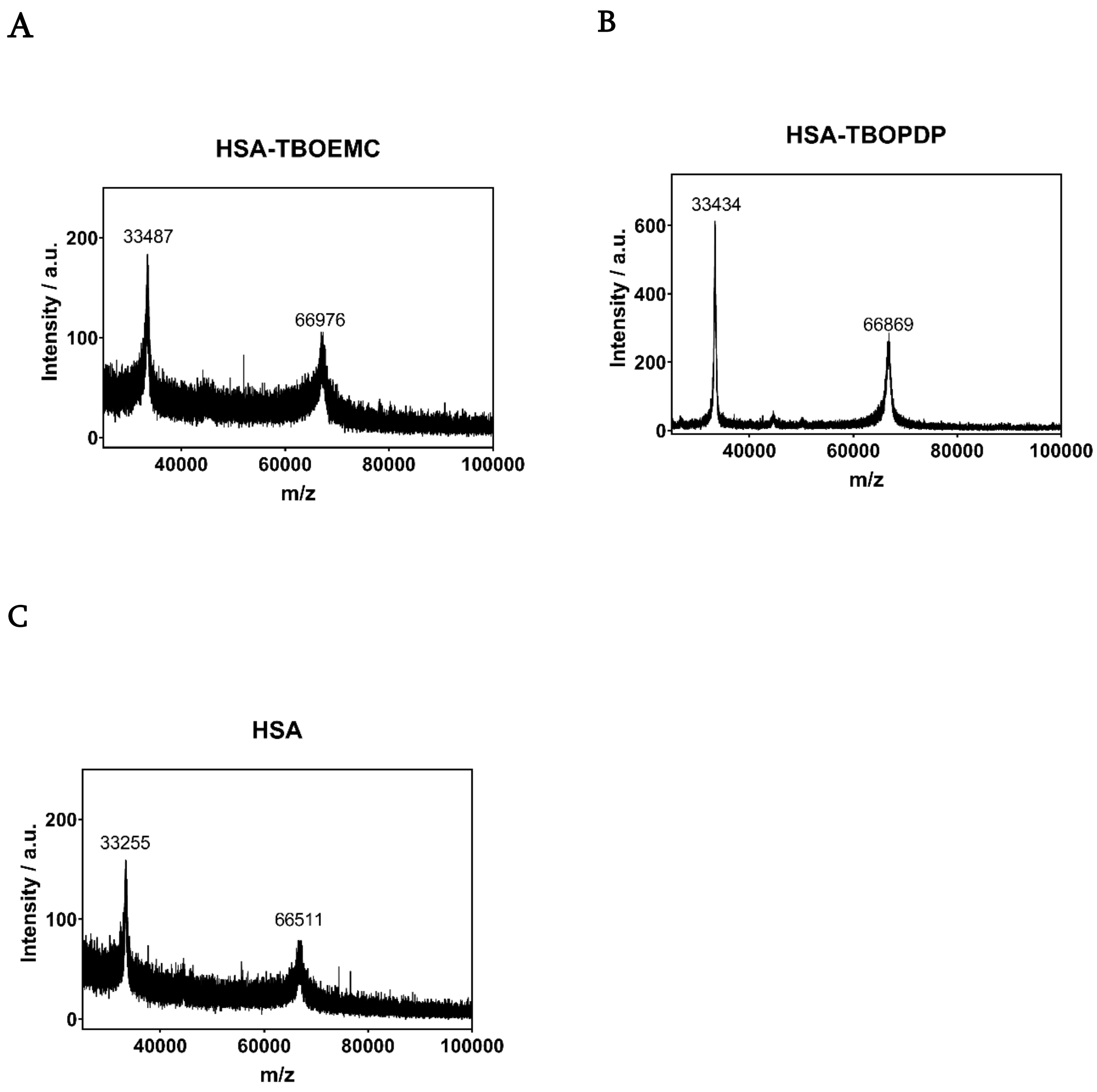

Figure S11. MALDI-TOF spectra: a) HSA-TBOEMC, b) HSA-TBOPDP, and c) HSA.[1]

1. From reference: Mariño-Ocampo, N.; Reyes, J. S.; Günther, G.; Heyne, B.; Fuentealba, D. Thiol-reacting toluidine blue derivatives: Synthesis, photophysical properties and covalent conjugation with human serum albumin. *Dyes and Pigments* 2022, 201.
